# Inhibition of the Growth of *Escherichia coli* and *Staphylococcus aureus* Microorganisms in Aesthetic Orthodontic Brackets through the In Situ Synthesis of Ag, TiO_2_ and Ag/TiO_2_ Nanoparticles

**DOI:** 10.3390/microorganisms12081583

**Published:** 2024-08-03

**Authors:** Paola Ariselda Sánchez Reyna, Oscar Fernando Olea Mejía, María G. González-Pedroza, Norma M. Montiel-Bastida, Bernabe Rebollo-Plata, Raúl A. Morales-Luckie

**Affiliations:** 1 Center for Advanced Studies and Research on Dentistry, Autonomous University of the State of Mexico (UAEMex), Toluca 50200, Mexico; pasanchezr@uaemex.mx (P.A.S.R.); nmmontielb@uaemex.mx (N.M.M.-B.); 2 Department of Materials Science, Center for Research in Sustainable Chemistry (CCIQS), Autonomous University of the State of Mexico (UAEMex), Km 14.5, Carr. Toluca-Atlacomulco, Toluca 50200, Mexico; ofoleam@uaemex.mx; 3 Department of Biotechnology, Faculty of Sciences, Autonomous University of the State of Mexico (UAEMex), Km 14.5, Carr. Toluca-Atlacomulco, Toluca 50200, Mexico; mggonzalezp@uaemex.mx; 4 Tecnológico Nacional de México, Instituto Tecnológico Superior de Irapuato, Carr. Irapuato-Silao Km 12.5, Irapuato 36821, Mexico; berebollo@itesi.edu.mx

**Keywords:** Ag, TiO_2_ and Ag/TiO_2_ nanoparticles, aesthetic orthodontic brackets of α-alumina, antibacterial effect

## Abstract

Plaque control is especially important during orthodontic treatment because areas of the teeth near brackets and wires are difficult to clean with a toothbrush, resulting in debris buildup of food or dental plaque, thus causing caries and periodontal disease. The objective of this study was to evaluate the antimicrobial properties of silver nanoparticles (AgNPs), titanium dioxide nanoparticles (TiO_2_NPs), and silver/titanium dioxide nanoparticles (Ag/TiO_2_NPs), synthesized on the surface of α-alumina ceramic brackets. The AgNPs and TiO_2_NPs were synthesized by a simple chemical method, and these were characterized by XRD, SEM, and XPS TEM; the antimicrobial activity was tested against *Staphylococcus aureus* and *Escherichia coli* by diffusion test. The results of this study demonstrated that by this simple chemical method, silver and titanium dioxide nanoparticles can be synthesized on the surface of α-alumina esthetic brackets, and these NPs possess good antimicrobial activity and the possibility of reducing dental caries, periodontal disease, and white spot generated during orthodontic treatment.

## 1. Introduction

Nanoscience is an emerging field that includes nanostructured materials, including the use of metallic nanoparticles such as Ag, Au, Ir, Pd, and Pt, which are used in different disciplines due to their multiple properties. In recent years, silver nanoparticles (AgNPs) have been one of the most broad-spectrum antibacterial materials used due to their high cytotoxicity in a wide range of microbial and fungal species, including antibiotic-resistant strains, and low toxicity in humans [1,2,3,4,5]. It has been possible to demonstrate that AgNPs can bind directly with the cell membrane of bacteria and then damaging or altering their functionality [4,6]. This is due to its high reactivity to substances such as proteins, enzymes, DNA, RNA, etc., because of interactions that occur against thiol functional groups, carboxylate, phosphate, hydroxyl, imidazole, indole, or amine [7,8,9]. These interactions can occur easily or combined which may cause a series of events that interfere microbial processes [10,11]. The factors that affect the toxicity of AgNPs are the particle size, since with smaller size, there will be greater toxicity, this being due to the surface area they present [12,13]; the shape of the nanoparticles also has to do with the properties that triangular nanoparticles can provide, for example, they have greater antibacterial activity than spherical ones [14]. There are many reports on the synthesis of AgNPs, and in all of them, one of the most important aspects that must be controlled is the precipitation of AgNPs from its precursor Ag+; and the second is the size or shape of the AgNPs after their formation. This is possible thanks to the synthesis method. Synthesis of AgNPs can be carried out by different methods: chemical reduction [15,16,17], photochemical reduction [18], gamma [19], electron beam [20,21], microwave [22,23], laser [24], ultra violet [25], electrochemistry [26], sonochemistry [27], and synthesis ultrasound [28,29,30].

TiO_2_ photoactive materials are used to sterilize different materials when irradiated with sunlight or ultraviolet light. TiO_2_ can inhibit microbial adhesion for its excellent photocatalytic activity [31]. By irradiating TiO_2_ with a UV light power greater than its bandwidth, an electron is promoted from the valence band to the conduction band; then, high-energy electron–hole pairs reacting with water or oxygen molecules form various reactive oxygen species (ROS), which are strong oxidizing agents that cause damage to the cell membrane and therefore cell death [32,33,34]. However, the effect is decreased in the dark, which limits their biomedical applications [35,36,37]. TiO_2_ nanoparticles (TiO_2_NPs) are anatase phase antibacterial photocatalysts that have attracted great interest for inactivation of bacteria, fungi, viruses, and even cancer cells [38,39,40,41,42]. Compared with regular particles, solids with nanometric TiO_2_ and high specificity morphological dimensions may offer a single reaction means for the construction of photoactive nanoscale devices due to its high specific surface. In recent years, great efforts have been devoted to the design and manufacture of nanostructured systems with tunable physicochemical properties for advanced photocatalytic applications [43,44,45]. In recent decades, many methods have been developed to synthesize TiO_2_NPs such as, for example, sol-gel, hydrothermal, solvothermal, microemulsion, etc. [46,47,48].

The combination of TiO_2_ and Ag provides an active photocatalytic semiconductor and a noble metal that can extend and promote applications through photochemistry and heterogeneous catalysis [49,50,51]. In fact, anchoring AgNPs as electron traps is favorable for the separation of photoelectrons and excited holes to improve the quantum efficiency of TiO_2_, which further helps in eliminating bacteria, so that a layer of TiO_2_ facilitates the dispersion of AgNPs, and therefore incorporating TiO_2_/Ag makes an excellent route to enhance the antibacterial behavior. The conventional method used to prepare Ag/TiO_2_ nanoparticles (Ag/TiO_2_NPs) is the sol-gel chemical reduction, thermal reduction, or photochemical reduction of silver ions. Several methods have been proposed for the Ag/TiO_2_ preparation: a single step sol-gel route [52], photoreduction of Ag^+^ in a suspension of TiO_2_ [53], and electrochemical deposition of AgNPs on the surface of TiO_2_ [54].

Currently, the use of antibacterial agents for the removal of dental plaque is limited, and anchoring is complicated, given the nature of the surfaces of some dental materials, for example elastomeric modules, resins, etc. [55,56,57,58]. However, in this work, we present the use of novel elements for the generation of nanoparticles, the effectiveness of using a bimetallic system, as well as a more specific and safe anchoring technique, which guarantees the antimicrobial effect, against two very representative microorganisms in bacterial models.

To verify the antibacterial effect of the materials generated against these microorganisms, a technique known as Kirby Bauer has been used, which is a microbiological technique that shows the halos of inhibition generated by the materials and the antibacterial activity of brackets decorated with nanoparticles. As has been seen, both AgNPs and TiO_2_NPs have certain antibacterial activity and together have a characteristic behavior. These particles were synthesized and supported on ceramic brackets manufactured from Al_2_O_3_, used in orthodontic treatment with fixed appliances. Their antimicrobial properties were characterized and evaluated, recognizing it as a potential system to reduce plaque accumulation and bacterial growth in orthodontic procedures.

## 2. Materials and Methods

Materials: the reagents were silver nitrate (AgNO_3_), sodium dodecyl sulfate (SDS), titanium dioxide (TiO_2_) 10 wt% dispersed in water, and hydrazine (N_2_H_4_ · H_2_O); all reagents were purchased from SIGMA-ALDRICH company, St Louis, MO, USA.

### 2.1. Synthesis and Incorporation of Nanoparticles in Brackets

#### 2.1.1. Ag Nanoparticles Supported on Brackets

AgNPs were synthesized in situ on a support of brackets of α-Al_2_O_3_, starting with a 20 mL solution of 10 mM of AgNO_3_ solution mixed with a 20 mL solution of SDS 10% for 30 min with magnetic stirring, and the mixture was subsequently added a solution N_2_H_4_ ·H_2_O (108 mM) as a reducing agent, followed by continued magnetic stirring for 30 min. Finally, the support with AgNPs was removed from the reaction mixture and washed with deionized water before being subsequently dried in an oven at 70 °C for 24 h.

#### 2.1.2. TiO_2_ Nanoparticles Supported on Brackets

Nanoparticles of TiO_2_ were anchored to the support of brackets of α-Al_2_O_3_: a 20 mL solution of TiO_2_ at 10 mM solution 10 wt% dispersed in water was mixed with a SDS 10% for 30 min with magnetic stirring; finally, the TiO_2_ nanoparticles support was removed from the reaction mixture and washed with deionized water to remove the excess, and then subsequently dried in an oven at 70 °C for 24 h.

#### 2.1.3. Ag/TiO_2_ Nanoparticles Supported on Brackets

The Ag/TiO_2_NPs on the support of brackets of α-Al_2_O_3_ were obtained as follows: 10 mL of TiO_2_ solution 10 mM 10% wt, dispersed in water, with a 10 mL of AgNO_3_ solution 10 mM was mixed, and to this mixture was added a 10% solution of 20 mL of SDS, before magnetically stirring for 30 min, then a N_2_H_4_ ·H_2_O (108 mM) solution was added as a reducing agent; subsequently, magnetic stirring continued for 30 min. Finally, the support with Ag/TiO_2_ nanoparticles was removed from the reaction mixture and washed with deionized water, and then subsequently dried in an oven at 70 °C for 24 h.

### 2.2. Characterization Techniques

The dry samples of 2 mL of silver, titanium dioxide, and silver/titanium dioxide nanoparticles were analyzed separately in a JEOL brand scanning electron microscope, microscope (Tokyo, Japan), with a JSM-6510LV Tungsten Filament coupled to an X-ray detector, for chemical analysis by OXFORD brand Energy Dispersion (EDS) (Atlanta, GA, USA), with a resolution of 137 eV.

For Transmission Electron Microscopy (TEM), the sample was analyzed using a drop of the nanoparticle solutions separately on Cu grids, and they were analyzed in a transmission electron microscope JEOL-2100 of 200 kV brand LaB6 filament (Tokyo, Japan) with a resolution of 0.23 nm point to point and 0.14 nm line by line. Micrographs were acquired digitally using a Gatan CCD camera, model SC200 (Pleasanton, USA).

Atomic Force Microscopy (AFM) VEECO Brand CP-II atomic force microscope (NY, USA: SPM Digital. Touch and touch modes have a 5 µm XY scan range and 1 µm Z scan range.

X-ray Diffraction (XRD) dry samples of the generated nanoparticles were analyzed in a D8 Advance Bruker LynxEye X-ray diffractometer (Karlsruhe, Germany) that has a copper anode X-ray source (Kα1 = 0.1540 Å).

For X-ray Photoelectron Spectrometry (XPS), the generated and dried nanoparticles were analyzed with a JEOL JPS-9200 X-ray photoelectron spectrometer (Tokyo, Japan); the computer uses a Mg Ka (hv = 1253.6 eV) X-ray source operated at 10 kV and 20 mA (200 W) and is equipped with a hemispherical electrostatic analyzer with a mean radius of 100 mm and a multichannel detector.

### 2.3. Antibacterial Activity

The antibacterial activity of the support with AgNPs, TiO_2_NPs, and Ag/TiO_2_ NPs was evaluated against both Gram-negative and Gram-positive bacteria, including species of *Escherichia coli* and *Staphylococcus aureus*, using the agar diffusion method [59]. The bacterial strains used in this study were acquired from the stock culture collection of the Biochemistry laboratory of the Faculty of Dentistry of the National Autonomous University of Mexico (UNAM). The strains were originally collected from clinical samples of the oral cavity of patients from the previously mentioned institution. These strains are native to central Mexico, having all been characterized through a set of culture media assays and biochemical tests. Luri Bertani (LB) and agar broth were used for growing *E. coli* and *S. aureus*. The endemic *E. coli* and *S. aureus* bacteria were inoculated in Petri dishes with Mueller Hinton agar and at a concentration of 1.5 × 10^8^ CFU/mL. The experiments were carried out as recommended by the Clinical and Laboratory Standards Institute [60]. The tests were performed in triplicate by placing a bracket previously treated with silver nanoparticles (AgNPs), titanium dioxide nanoparticles (TiO_2_NPs), and silver nanoparticles/titanium dioxide (Ag/TiO_2_NPs) separately and a control bracket (without treatment) at 37 °C for 24 h.

## 3. Results and Discussion

### 3.1. Elemental and Superficial Characterization of the Al_2_O_3_ Bracket

**Figure 1:**
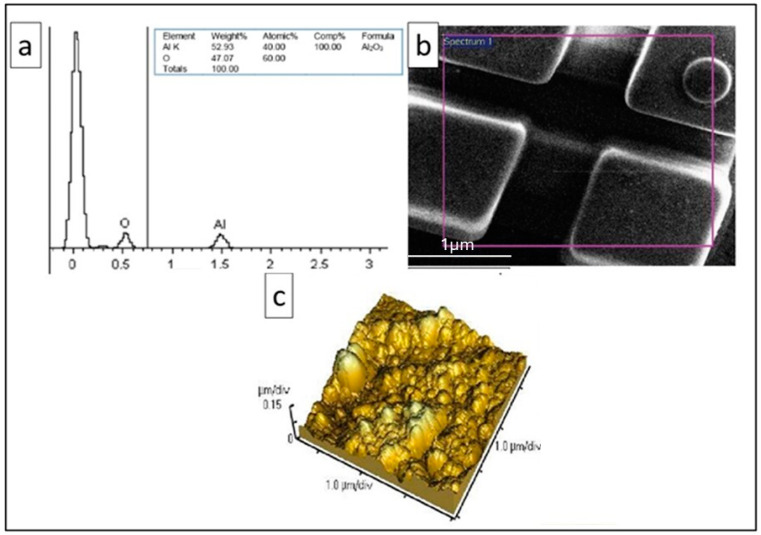
Microscopic and elemental characterization: (**a**) SEM-EDS elemental characterization; (**b**) micrograph of the bracket; and (**c**) AFM micrograph of the bracket surface.

The elemental analysis by EDS, shown in Figure 1a, carried out on the surface of the bracket shows only the presence of oxygen and aluminum, and the atomic percentage reported is 60% for oxygen and 40% for aluminum; this stoichiometric relationship corresponds to aluminum oxide as can be seen, inferring the chemical composition of Al_2_O_3_. Figure 1b clearly shows the surface of the bracket, where a typical morphology of a ceramic bracket is observed. The bracket Al_2_O_3_ support was characterized for AFM where the topography of a polycrystalline material can be observed, with an average grain size of 400 nm; it has high roughness, and in Figure 1c, it is observed that the grain size is not homogeneous and a difference between the peaks and valleys is perceived.

**Figure 2:**
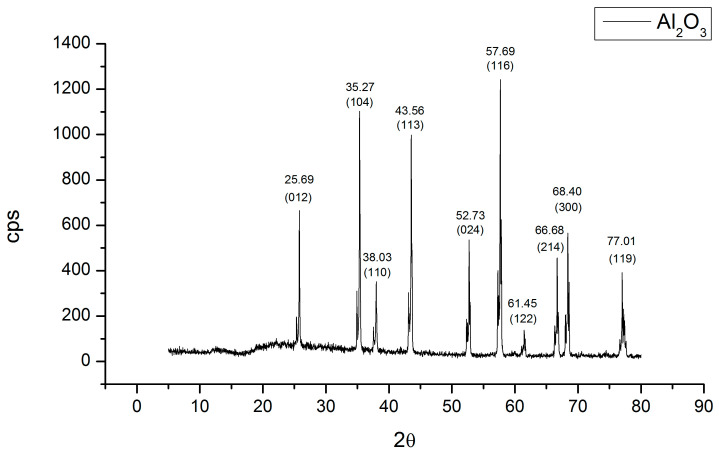
XRD pattern of the support (bracket). The peaks were compared with JCPDS-ICCD-46-1212 card, corresponding to α-Al_2_O_3_.

Figure 2 shows the XRD diffractogram that was compared with the JCPDS-ICCD-46-1212 card, indicating that the Al_2_O_3_ is in the alpha phase. In the diffractogram, you can see the presence of a small peak to the left of the main diffraction, 2θ~25.6°; to the left, this small peak appears with an intensity of a quarter of the main peak, and thus it is repeated. In the remaining diffractions, since the surface of the bracket is not flat, it presents different heights in its topography, and this causes the appearance of this double peak at 52.7° and 57.6° due to the difference in the distance of the topography of the material.

### 3.2. Morphological Characterization of the Synthesized Nanoparticles

**Figure 3:**
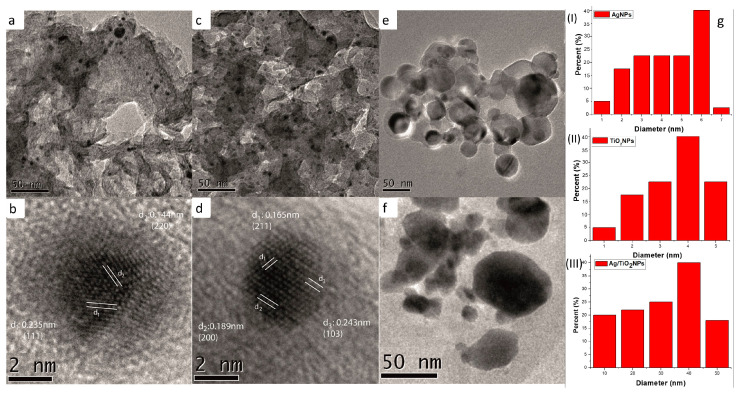
TEM micrographs: (**a**) Ag nanoparticles; (**b**) high resolution analysis of Ag nanoparticles; (**c**) TiO_2_ nanoparticles; (**d**) high resolution analysis of TiO_2_ nanoparticles; (**e**,**f**) Ag/nanoparticles TiO_2_. (**g**) Nanoparticle size histograms; (I) AgNPs, (II) TiO_2_NPs, and (III) Ag/TiO_2_NPs.

The synthesized AgNPs were characterized using the TEM technique to obtain information on the particle size and distribution of Ag deposited on the surface of the Al_2_O_3_ supports. As shown in the micrographs in Figure 3a,b,e,f, the presence of AgNPs, TiO_2_NPs, and Ag/TiO_2_NPs is corroborated, all being quasi-spherical. Regarding the HRTEM analysis where the information on the interplanar distance of the AgNPs is shown, this being 0.235 nm and 0.144 nm, respectively in the (111) and (220) planes, consistent with the JCPDS 04-0783 card. We observe, in Figure 3g(I), a distribution of AgNPs with a mean size of 4.2 nm on the surface of the Al_2_O_3_ bracket.

Likewise, the HRTEM technique was used to obtain information on the interplanar distance of the TiO_2_NPs, these being 0.243 nm, 0.189 nm, and 0.165 nm corresponding to (103), (200), and (211), respectively, according to the JCPDS card 021-1272. TiO_2_NPs with a mean size of 3.6 nm are observed on the surface of the bracket. In Figure 3e,f, a small number of titanium dioxide nanoparticles with an average size of 31 nm were deposited on the surface of the Al_2_O_3_ support. It is also observed that the silver nanoparticles are deposited on the surface of the individual TiO_2_ crystallites; this can be observed in Figure 3f depending on the difference in shades.

### 3.3. Characterization of the Nanoparticles Supported on the Surface of the Al_2_O_3_ Brackets

**Figure 4:**
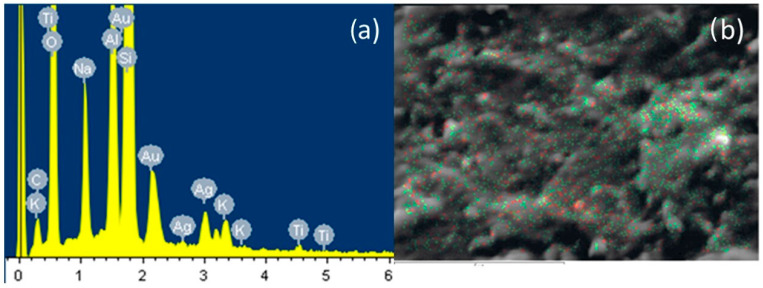
Analysis by SEM-EDS. (**a**) Spectrum of the elemental analysis of Ag/TiO_2_ NPs, (**b**) elemental mapping; Ti (Red), Ag (Green) on surface characterization of the nanoparticles supported on the surface of the brackets.

Figure 4a shows the elemental composition of the surface of the Ag/TiO_2_NPs deposited on the bracket as determined by EDS, where the presence of aluminum, titanium, silver, and oxygen is mainly observed; these samples were coated with gold. As seen in Figure 4b, the mapping of the distribution of the nanoparticles is homogeneous, with the Ag concentration being higher. Therefore, it can be assumed that the Ag/TiO_2_NPs were deposited on the Al_2_O_3_ surface support.

**Figure 5:**
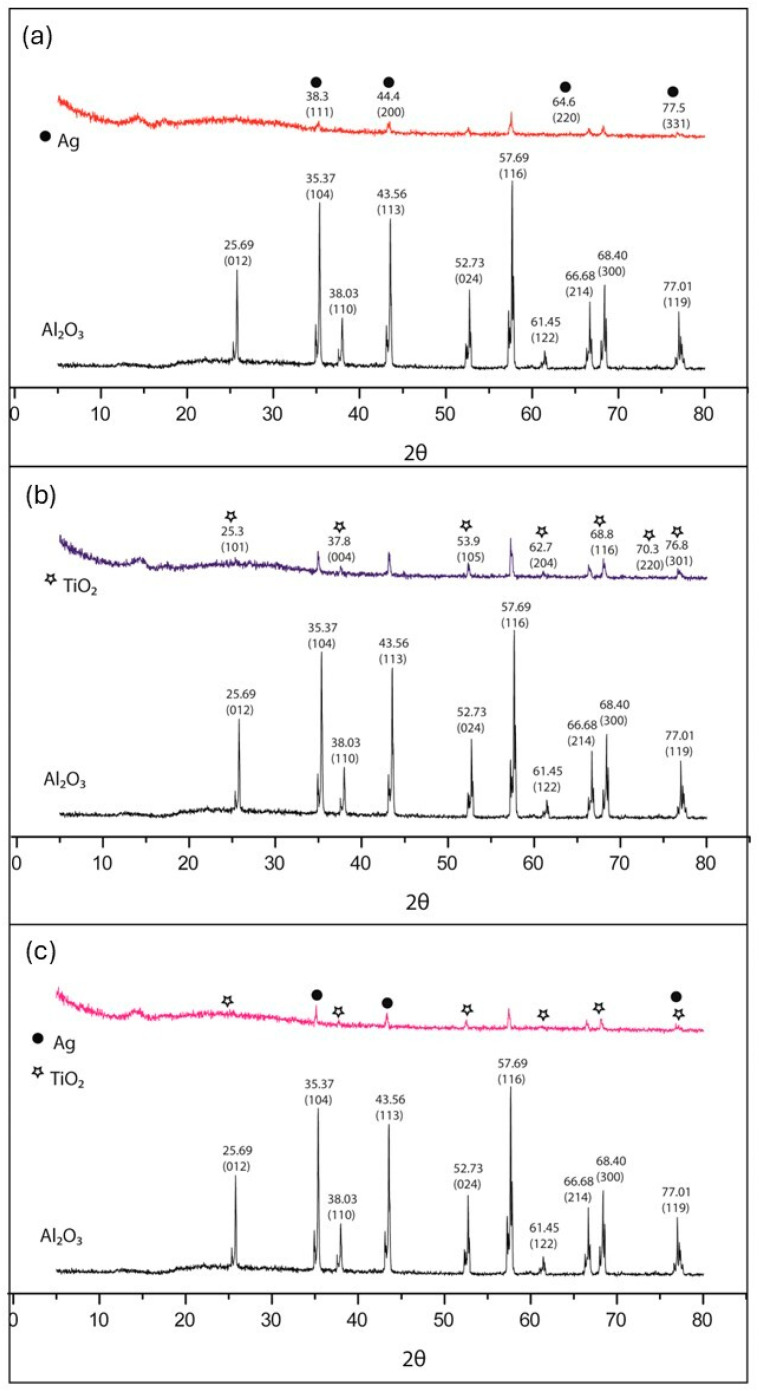
X-ray diffraction (XRD). (**a**) XRD pattern of AgNPs anchored Al_2_O_3_ support. (**b**) XRD pattern of TiO_2_NPs anchored Al_2_O_3_ support. (**c**) Pattern X-ray diffraction of the Ag/TiO_2_NPs anchored Al_2_O_3_ support.

In Figure 5a, the pattern of X-ray diffraction of AgNPs deposited on the support surface Al_2_O_3_, shows that the peaks at 38.3, 44.4, 64.6, and 75.5 of AgNPs coincide with the peaks of Al_2_O_3_; we observed that they are less intense, so we assume that the support surface was covered by AgNPs. Also, in Figure 5b, the pattern of X-ray diffraction of Ag/TiO_2_ NPs deposited on the support surface of Al_2_O_3_ shows that the peaks at 23.3°, 37.8°, 53.9°, 62.7°, 68.8°, 70.3°, and 76.8° of TiO_2_NPs match those shown by Al_2_O_3_ but are less intense, so we assume that the support surface was covered by NP’s TiO_2_.

In Figure 5c, one can see the pattern of X-ray diffraction of the combination of Ag/TiO_2_NPs deposited on the support surface Al_2_O_3_ shown that peaks at 38.3°, 44.4°, and 75.5° of Ag NPs and peaks at 23.3°, 37.8°, 53.9°, 62.7°, 68.8°, and 76.8° of the TiO_2_NPs match those of Al_2_O_3_; both materials are deposited individually, making properties make the most of materials. The characterization technique is consistent with the analysis on elemental chemical mapping, where this distribution was observed. The diffractograms show that the peaks associated with the alumina decrease with the increment in the concentration of Ag and TiO_2_ [61,62,63].

**Figure 6:**
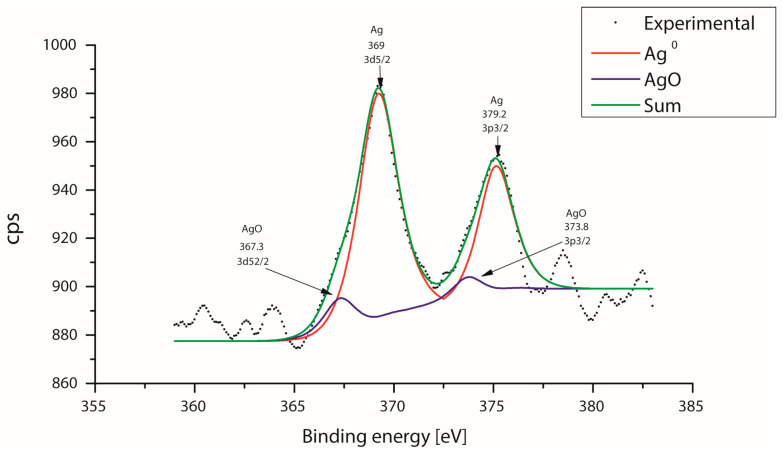
XPS spectra of AgNPs on Al_2_O_3_ support.

XPS spectra were obtained after etching with Ar^+^ for 10 s. The C 1s (binding energy, BE = 285.00 eV) was used as a reference for calibrating binding energy in all spectra of the samples. For the sample, AgNPs were analyzed by XPS to determine the oxidation state of the Ag. Figure 6 shows the XPS spectrum and corresponding oxidation states of the silver deconvolutions, indicating mostly the presence of metallic silver, as it can be seen that a large part of the silver is metallic while a small contribution is also observed assigned to Ag-O; the above indicates the almost total reduction of silver ions which confirms the effectiveness of the reduction method proposed for the formation of silver nanoparticles from the silver (Ag^+1^) ions.

**Figure 7:**
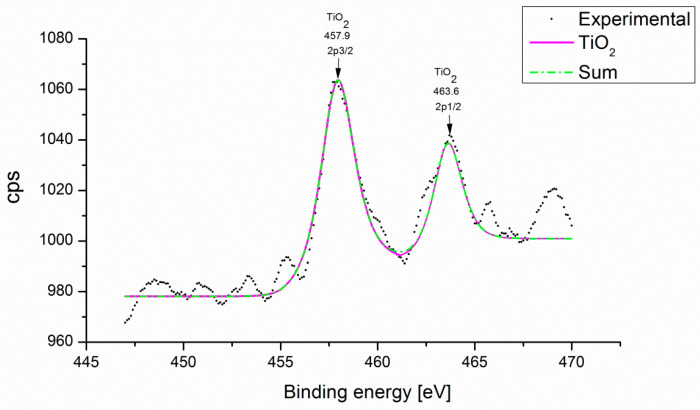
XPS Spectra TiO_2_NPs on Al_2_O_3_ support.

In Figure 7, the Ti deconvolution was performed, and there is only one signal corresponding to Ti-O, which confirms the effectiveness of the proposed reduction method to anchor the titanium dioxide nanoparticles and the generation of titanium dioxide nanoparticles. Because the XPS is highly sensitive to the surface, the AgNPs are exposed largely on the surface and cover a portion of the surface of the TiO_2_ particles; as observed in Ag3d_5/2_ signals and Ti2p_3/2_, they can be comparable in magnitude despite having different concentrations. This argument is consistent with the morphological characteristics of the samples [64,65,66,67].

Dodecyl-sulfate (DS) surfactant molecules were used in 10 wt% to provide a hydrophobic character to the photocatalyst particles and increase the dispersibility of the originally hydrophilic photocatalyst particles in the polymer film [68]. The mechanism of anchoring the NPs to the surface of the alumin is described with respect to the high electronic density of the oxygen of the alumina that electrostatically attract the NPs, and there is also a mechanical retention due to the roughness of the surface of the bracket.

### 3.4. Antimicrobial Activity

**Figure 8:**
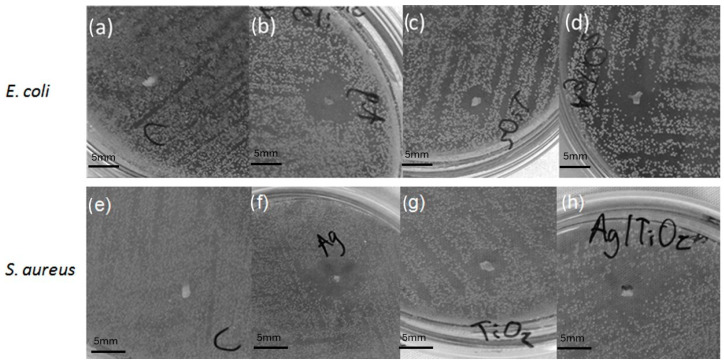
It shows the inhibitory halos of brackets (mm) on *E. coli*: (**a**) bracket without nanoparticles; (**b**) bracket with AgNPs; (**c**) bracket with TiO_2_NPs; (**d**) bracket with AgNPs, TiO_2_NPs, and Ag/TiO_2_NPs. It shows the inhibitory halos of brackets on *S. aureus*: (**e**) bracket without nanoparticles; (**f**) bracket with AgNPs; (**g**) bracket with TiO_2_NPs; (**h**) bracket with Ag/TiO_2_NPs.

In the microbiological testing, the samples prepared with AgNPs, TiO_2_NPs, and Ag/TiO_2_NPs on brackets exhibited a bactericidal effect, which can be observed by formation of an inhibitory halo. This occurs in both microorganisms *E. coli* and *S. aureus*, Gram (−) and Gram (+), respectively; this can contrast the inhibitory halo in Figure 8, with respect to the control bracket. This is consistent with several investigations where *E. coli* inactivation is mainly due to the destruction of the cell wall by Ag and TiO_2_ [69,70].

In Figure 8, the antimicrobial activity in *E. coli* of AgNPs, TiO_2_ NPs, and Ag/TiO_2_NPs and a control bracket is shown, and it is shown in Figure 8b,d that both AgNPs and Ag/TiO_2_NPs have a similar inhibitory halo, while in Figure 8c, it can be seen that TiO_2_NPs presents a significant inhibition halo, all compared to the control non-inhibitory halo presented as shown in Figure 8a. As for the antimicrobial activity in *S. aureus* of AgNPs, TiO_2_NPs, and Ag/TiO_2_NPs and a bracket control, it is observed that in Figure 8f, the AgNPs have a greater inhibitory halo than the TiO_2_NPs inhibitory halo in Figure 8g, and in Figure 8h, the Ag/TiO_2_NPs have an inhibitory halo slightly higher than those of AgNPs and TiO_2_NPs alone, all compared to the control bracket which did not present an inhibitory halo, as shown in Figure 8e (Table 1). In general, all treatments had a greater effect against *E. coli* bacteria than *S. aureus*.

This difference in bacterial resistance of Gram-negative and Gram-positive bacteria agrees with previously published research [71,72,73]. The tolerance of Gram-negative bacteria to different nanoparticles could be attributed to the layer of lipopolysaccharide (LPS), providing an external bacteria cell membrane. This LPS layer not only contributes to cell integrity but also hinders the absorption of Ag^+^ and/or AgNPs [74]. It is unclear whether this trend is universal for all species of Gram-negative and Gram-positive bacteria [75]. It is important to highlight that the Ag/TiO_2_NPs increase considerably in size; according to the literature, we expected to obtain greater antibacterial activity; however, they are almost similar. This may be due to the arrangement of the atoms in the system according to the atomic radii of each of the elements or because when the system dries, it agglomerates, and the size of the supported nanoparticle becomes larger. Other authors have shown that the antimicrobial activity on other types of materials, such as wires used in orthodontics, show a better antibacterial effect with smaller silver nanoparticles [76], and even with other methods of synthesis of titanium dioxide nanoparticles, such as N-doped TiO_2_ thin films coated on stainless steel brackets under visible light irradiation, an important antibacterial effect was achieved, as well as the results obtained in this work using a simpler method [77].

According to the literature, we can infer that the mechanism of action of the metallic nanoparticles is the following: nanoparticles can bind directly to the cell membrane of bacteria and then damage or alter its functionality. This is due to its high reactivity to substances such as proteins, enzymes, DNA, RNA, etc. [7,8,9].

## 4. Conclusions

In this study, synthesis of AgNPs, TiO_2_NPs, and Ag/TiO_2_NPs on the surface of Al_2_O_3_ was performed by a simple, inexpensive chemical reduction method. These nanoparticles are quasi-spherical, with defined sizes that offer different behavior alone and in combination. In the microbiological testing, an inhibitory effect was achieved in all samples, having a greater inhibitory halo in samples of Ag and Ag/TiO_2_ than TiO_2_ in the samples of both bacteria. Finally, these results show that supporting Ag and Ag/TiO_2_NPs in brackets can be an alternative to avoid generating stains on teeth during orthodontic treatment; in future work, it will be necessary to carry out more specific tests that simulate dentobacterial plaque until treatment is assured in patients.

## Figures and Tables

**Table 1:** Results of diffusion tests on disk.

	Diameter of Inhibition Halos (mm)			
Strain	Control	Ag	TiO_2_	Ag/TiO_2_
*S. aureus*	NP	8.8 ± 0.3	6.0 ± 0.6	8.7 ± 0.8
*E. coli*	NP	8.3 ± 0.2	5.0 ± 0.3	8.9 ± 0.1

Results are represented by the mean ± standard error.

## Data Availability

The original contributions presented in the study are included in the article, further inquiries can be directed to the corresponding author.
